# Discovery of a Novel Jingmenvirus in Australian Sugarcane Soldier Fly (*Inopus flavus*) Larvae

**DOI:** 10.3390/v14061140

**Published:** 2022-05-25

**Authors:** Agathe M. G. Colmant, Michael J. Furlong, Kayvan Etebari

**Affiliations:** 1 Unité des Virus Émergents (UVE: Aix-Marseille Univ-IRD 190-Inserm 1207), 13005 Marseille, France; agathe.colmant@uq.net.au; 2 Australian Infectious Diseases Research Centre, School of Chemistry and Molecular Biosciences, The University of Queensland, Brisbane, QLD 4072, Australia; 3 School of Biological Sciences, The University of Queensland, Brisbane, QLD 4072, Australia

**Keywords:** soldier fly, sugarcane pest, virome

## Abstract

In Australia, soldier flies are major pests of sugarcane, and they can cause significant yield losses in some areas, possibly due to the virus’ transmission to the plants. We sequenced fly larvae salivary glands and identified a novel jingmenvirus, putatively named Inopus flavus jingmenvirus 1 (IFJV1). Phylogenetic trees confirmed that IFJV1 groups with insect-associated jingmenviruses, newly identified flavivirus-like viruses with a segmented genome. After the design and the validation of molecular detection systems for IFJV1, larval homogenates were passaged on insect and vertebrate cells, but IFJV1 could only be detected in the first two passages in insect cells and not at all in vertebrate cells. Despite this lack of consistent replication in laboratory models, this virus does replicate in its host *Inopus flavus*, as sequenced, small RNA from the larvae matched the IFJV1 sequences. Moreover, they were found to be predominantly 21 nucleotides long and map to the whole sequences on both strands, which is typical of an actively replicating virus. This discovery confirms the worldwide presence of jingmenviruses which, until now, had only been detected on four continents. However, the study of IFJV1 tropism and the possible pathogenicity to its host or the sugarcane it parasitizes requires the development of a stable replication model.

## 1. Introduction

Sugarcane soldier flies are important pests, as they cause significant yield losses in some sugarcane regions in Australia. Soldier flies represent a species complex that comprises at least six endemic species that are economically important pests of sugarcane [1]. The damage caused by one of the species, *Inopus flavus* (Diptera: Stratiomyidae), has become more obvious recent years in Australia, even though its distribution is believed to be restricted to eastern–central Queensland [2,3]. Soldier fly pest management is difficult in sugarcane crops as insecticides are ineffective and the varietal tolerance to larval feeding is limited. Small numbers of larvae can cause significant damage to the plant and reduce the crop yields. We aimed to investigate whether this effect is linked to soldier fly larvae transmitting viruses to the plant during feeding. Moreover, insect RNA viruses are capable of causing a significant reduction in the field populations of agricultural and forestry pests. There are several examples of using insect-specific RNA viruses as biological control agents, sometimes in combination with genetically engineered crops [4]. In the context of this study, novel viruses are, therefore, of particular interest due to their potential as plant or insect pathogens, which would then need to be either managed or could be harnessed as biological control agents. The advent of next-generation sequencing (NGS) technology has created a great opportunity for novel virus discoveries.

Jingmenviruses are a group of novel positive single-stranded RNA (+ssRNA) viruses, currently designated by the ICTV as an unclassified sub-genus in the *Flavivirus* genus and *Flaviviridae* family, which include viruses of medical and veterinary importance, such as tick-borne encephalitis or dengue viruses. The first jingmenvirus identified as such was Qin et al.’s 2014 discovery of the Jingmen tick virus (JMTV) in *Rhipicephalus microplus* ticks collected in China [5]. JMTV has a four-segmented genome. The first and third segments present one open reading frame (ORF) each, which code for non-structural proteins 1 and 2, respectively, similar to the NS5 (RNA-dependant-RNA-polymerase (RdRp) and methyltransferase) and NS2B/NS3 (serine protease and helicase) flavivirus proteins. Segments 2 and 4 code for two structural proteins each (VP4 and VP1; VP2 and VP3, respectively) and are more genetically distant from flaviviruses than the non-structural proteins. Since this discovery, several other virus species associated with ticks or human infections (i.e., Alongshan virus, Yanggou tick virus, Xinjiang tick virus 1, and Takashi virus), other vertebrates (i.e., bats and rodents), insects, or plants [6,7,8,9,10,11] have been discovered. To date, jingmenviruses have been detected in a wide range of hosts and in geographical locations on four continents (specifically Asia, America, Africa and Europe). Very few reports describe isolation attempts, particularly for insect-associated viruses, while the replication of the prototype strain of the prototype jingmenvirus JMTV could only be detected for a couple of passages on vertebrate (DH82, dog) and insect (C6/36, mosquito) cell lines, or in intracranially injected newborn mice [5]. Isolation has been attempted for other strains of this virus, but all have been unsuccessful or limited to the first couple of passages [8,12,13,14,15,16,17].

The identification of new insect viruses and further investigation into their impact on soldier fly populations in different regions will provide a better understanding of the potential interactions between insect-specific viruses and their hosts, which could potentially lead to the identification of new biological control agents.

## 2. Materials and Methods

### 2.1. Sample Collection and RNA Extraction

Sugarcane yellow soldier fly (*Inopus flavus*) larvae were collected from an infested sugarcane field near Hay Point, Queensland (21°18′5″ S, 149°14′7″ E) in 2019. Sugarcane stools were excavated from the ground and large larvae were manually collected from the roots and the associated soil. Larvae were transported to the University of Queensland’s laboratory for RNA extraction and next-generation sequencing. The approach was unbiased, since no attempt was made to enrich viral particles through filtration, centrifugation, or nuclease treatment. The total RNA samples were extracted from the larvae’s salivary glands, as previously described in Etebari et al. 2020 [2]. Briefly, the larval body surfaces were disinfected by being soaked in 75% ethanol for 30 s and rinsed in phosphate-buffered saline (PBS). The salivary glands (SGs) were then extracted by pulling out the head capsule and removing all other tissues, such as fat body droplets. The SG tissues were pooled and transferred to a Qiazol lysis reagent for RNA extraction, according to the manufacturers’ instructions (QIAGEN, Hilden, Germany; Cat No.: 79306), in pools of 20. Six pools were sent for total RNA sequencing on a HiSeq 4000 (Illumina, San Diego, CA, United States) by the Australian Genome Research Facility (AGRF, Melbourne), after DNase treatment and an RNA quality control check. Deep sequencing raw data were deposited in the National Centre for Biotechnology Information’s (NCBI’s) Gene Expression Omnibus (GEO) and are accessible through the GEO series accession number: GSE127658.

### 2.2. Transcriptome Data Analysis and Virus Discovery

In this study, the CLC Genomics Workbench version 20.0.1 (Qiagen, Hilden, Germany) was used for bioinformatics analyses. All libraries were trimmed from any remaining vector or adapter sequences. The reads were 75 bp pair-ended, with an average fragment size of 350 bp and an insert size of 230 bp. Low-quality reads (i.e., a quality score below 0.05) and reads with more than two ambiguous nucleotides were discarded. As the *I. flavus* genome is not sequenced, all reads were mapped to the black soldier fly genome, *Hermetia illucens* (the closest available relative), which served as a proxy genome reference (GCF_905115235.1) to remove insect-related reads. Unmapped reads were retained for de novo assembly and potential virus discovery.

Contigs were constructed with a kmer size of 45, bubble size of 50, and a minimum length of 500 bp and were then corrected by mapping all the reads against the assembled sequences (min. length fraction = 0.9 and maximum mismatches = 2). The generated contigs were compared with the NCBI viral database, using local BLAST and BLASTx algorithms. The e-value was set to 1 × 10^−10^ to maintain a high sensitivity and a low false-positive rate. To obtain the segment 2 sequence, the contigs were compared with a database comprising all available sequences from jingmenvirus segments with a glycoprotein (segment 4 for the following mosquito associated-jingmenviruses: the Guaico Culex virus and the Mole Culex virus, and segment 2 for all other jingmenviruses). 

Putative jingmenvirus sequences were re-mapped to the RNA-Seq data to inspect for sufficient coverage and possible mis-assembly. The CLC Genomic Workbench’s RNA-Seq function (min. length fraction = 0.9, maximum mismatches = 2, insertion cost = 3, and deletion cost = 3) on a non-strand-specific option was used. 

The signal peptide, the potential glycosylation sites, and the transmembrane domains were predicted using the online tools SignalP 6.0 (https://services.healthtech.dtu.dk/service.php?SignalP), NetNGlyc (https://services.healthtech.dtu.dk/service.php?NetNGlyc-1.0), and TMHMM (https://services.healthtech.dtu.dk/service.php?TMHMM-2.0), accessed on 22 January 2022 [18].

### 2.3. Phylogenetic Analysis

The NSP1 and NSP2 amino acid (aa) sequence of both tick- and insect-associated jingmenviruses, including those of IFJV1, were used to build phylogenetic trees. First, multiple aa sequence alignments were performed with MUSCLE using Geneious Prime version 2022.0.2 (Auckland, New Zealand). Then, the maximum-likelihood phylogenetic trees were inferred using a JTT substitution matrix and assumed a discretized gamma rate distribution with four rate categories (shape parameter fixed at 1.0) and with 1000 bootstraps.

### 2.4. In Vitro Isolation Attempts 

The samples used for the isolation attempts were whole larval body homogenates that corresponded to the positive salivary glands and were homogenized in 500 μL phosphate-buffered saline (PBS, pH 7.4) with or without the addition of phenylthiourea (PTU) at saturation, to prevent melanization. The whole larvae bodies were used in order to maximize the chances of isolating the newly identified virus, since replicating viruses are present in higher titers in the bodies of their insect hosts than in their salivary glands.

The cell lines used for the isolation attempts were C6/36 *Aedes albopictus*-derived cells, maintained at 28 °C in RPMI 1640 with 2–5% fetal bovine serum (FBS); S2 *Drosophila melanogaster*-derived cells maintained at 28 °C in Schneider’s drosophila medium with 10% FBS; BSR baby hamster kidney *Mesocricetus auratus*-derived cells, and Vero African green monkey kidney *Cercopithecus aethiops*-derived cells, maintained at 37 °C with 5% CO2 in DMEM and with 5% FBS. All cell lines are available at School of Biological Sciences, The University of Queensland. All cell culture media were supplemented with 50 U/mL of penicillin, 50 μg/mL of streptomycin, and 2 mmol/L of l-glutamine.

Cells were seeded with 1 × 10^5^ cells per well in 24-well plates and incubated overnight at 28 °C or 37 °C for insect and vertebrate cells, respectively. The cell culture’s supernatant was removed and replaced with 200 µL of inoculum (homogenate diluted 1/10, filtered through a 0.22 µm sterile filter). The cells were left to incubate at room temperature for 30 min on a rocker and for a further 60 min at 28 °C (insect) or 37 °C (vertebrate). After incubation, the inoculum was removed and 50 µL was reserved for RNA extraction. The cells were washed three times with 750 μL of sterile phosphate-buffered saline (PBS) and the cell culture medium was replenished with 750 µL of fresh medium with 2% FBS. These cultures were left to incubate for 7 days at 28 °C or 37 °C for insect and vertebrate cells, respectively. After incubation, RNA was extracted from the harvested supernatants, which were also passaged on freshly seeded cells. The RNA extractions were performed using the Machery Nagel Nucleospin RNA extraction kit, following the manufacturer’s instructions. The RNA extracts were used as templates with the Superscript III One-Step RT-PCR System, the Platinum Taq DNA polymerase kit (Invitrogen) (following the manufacturer’s instructions), alongside primers designed to detect the jingmenvirus segments (see Table 1). The RT-PCR results were visualized by agarose gel electrophoresis.

### 2.5. Viral Derived Small RNA Analysis 

To analyze the host RNAi’s response to IFJV1 and to determine whether the virus was replicating in its host, a small RNA (sRNA) library was generated from one of the positive pools of 20 SG from starved individuals, using the NEBNext^®^ Multiplex Small RNA Library Prep Kit for Illumina^®^, at the Novogene Genomics Singapore Pte Ltd (Singapore). The purified cDNA libraries were sequenced on a Novaseq 6000 (SE50), and raw sequencing reads were obtained using Illumina’s Sequencing Control Studio software v1.7. Raw data were stripped of adapters, and reads with a quality score above 0.05 and with less than two ambiguous nucleotides were retained. Reads without 3′ adapters and reads with less than 16 nt were discarded. The clean reads were mapped to each IFJV1segment. We examined both the size distribution of the viral-derived sRNA fragments, as well as the genomic distribution for each segment on both strands (positive- and negative-sense).

## 3. Results

### 3.1. Identification of Inopus Flavus Jingmenvirus 1

**Figure 1:**
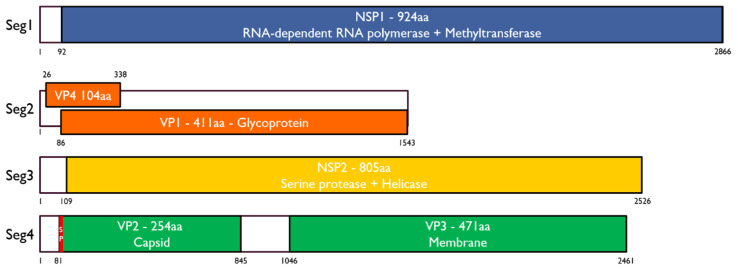
Genome organization of IFJV1. Segments are represented as black boxes with open reading frames highlighted in colors. The signal peptide of VP2 in segment 4 is represented in red. SP: signal peptide; aa: amino acid. Genbank accession numbers: OM869459-OM869462.

We identified several RNA viruses from the RNAseq libraries of salivary glands from six pools of 20 SG from soldier fly larvae, collected from north Queensland, Australia, in 2019. Here, we will focus only on a novel jingmenvirus we discovered and putatively named Inopus flavus jingmenvirus 1 (IFJV1). The sequences obtained for IFJV1 cover the putative full coding sequences of the four segments. The organization of the IFJV1 genome follows what has been found previously for insect-associated jingmenviruses (Figure 1). Segment 1 codes for NSP1—a 924 aa-long non-structural protein with RdRp and methyltransferase domains. Segment 2 is bicistronic and codes for the 485 aa-long glycoprotein (VP1) with four transmembrane domains and the putative 104 aa-long structural protein (VP4) with two transmembrane domains. Segment 3 codes for the 805 aa-long second non-structural protein (NSP2), which contains serine protease and helicase domains. Segment 4 is bicistronic and codes for two 254 and 471 aa-long structural proteins (VP2 and VP3). VP2 has a signal peptide with a predicted cleavage site between positions 16 and 17 and no predicted N-glycosylation sites, while VP3 contains at least six transmembrane domains. These proteins are the putative equivalent of the capsid and membrane proteins found in flaviviruses. The IFJV1 genomic sequences were deposited on Genbank and have been assigned the following accession numbers: OM869459-OM869462.

### 3.2. Phylogenetic Analysis

**Figure 2:**
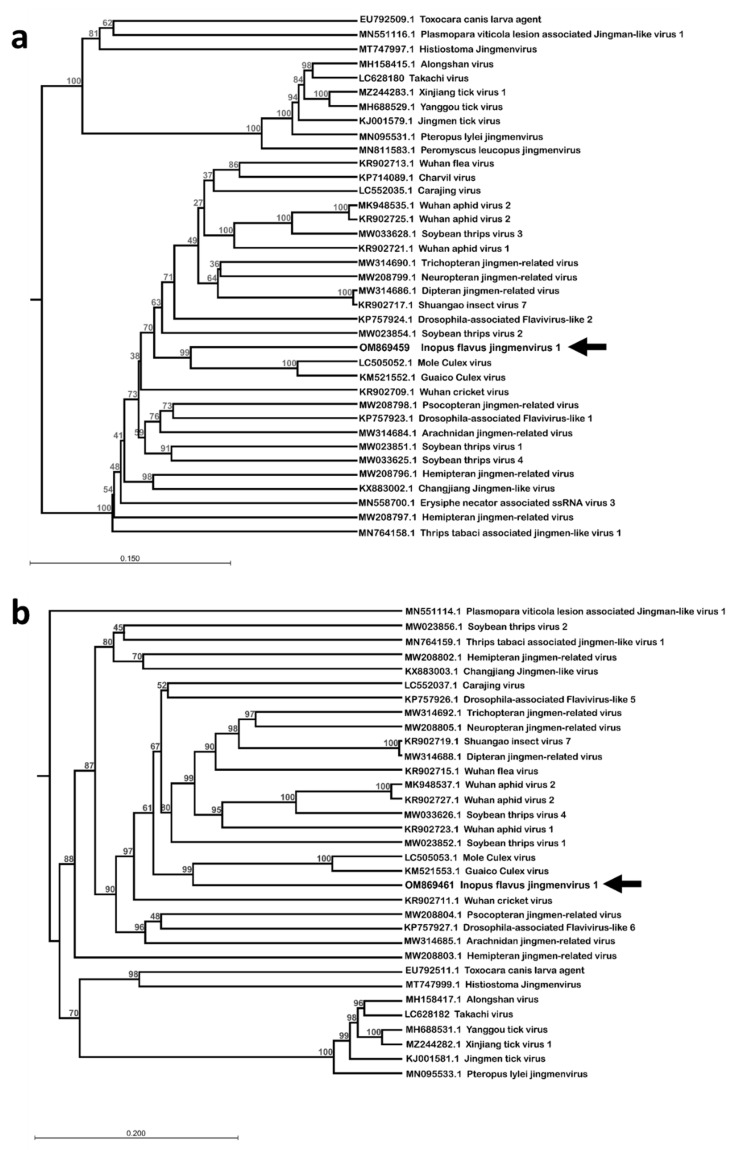
Phylogenetic analysis of the amino acid sequence of NSP1 (**a**) and NSP2 (**b**) of Inopus flavus jingmenvirus 1, aligned with reference jingmenviruses. These maximum-likelihood phylogenies were inferred using a JTT substitution matrix and assumed a discretized gamma rate distribution with four rate categories and with 100 bootstraps. Bar: branch length. The identified virus in this study is shown with an arrow.

The ORF aa sequences were compared with published sequences using NCBI BLAST with the blastp algorithm. According to these comparisons, IFJV1 is most similar to the Mole Culex virus and the Guaico Culex virus, two insect-associated jingmenviruses isolated from mosquitoes [17,19]. Their NSP1 share around 50% aa identity, their structural proteins VP2 and VP3 share approximately 25% aa identity, and their NSP2 share 40% aa identity. These identity percentages leave no doubt that IFJV1 is indeed a novel species of virus. The clustering of IFJV1 sequences with the mosquito-associated jingmenviruses within the insect-associated clade of jingmenviruses was confirmed by phylogenetic analysis on the aa sequences of NSP1 and NSP2 (Figure 2). The phylogenetic analyses were only performed on these two segments, as their organization is conserved for all known jingmenviruses, while the organization of the ORFs of segments 2 and 4 varies.

### 3.3. In Vitro Virus Isolation

Two homogenates from the bodies of soldier fly larvae with IFJV1-positive salivary glands were passaged on insect cell lines, specifically, C6/36 mosquito- and S2 drosophila-derived cells, in an attempt to isolate the virus. While IFJV1-specific amplicons could be amplified from the RNA from the inoculum and the first two passages, no amplicon was detected at the third passage, suggesting that the cells were not able to stably support the viral replication. A similar attempt was made on vertebrate BSR hamster- and Vero monkey-derived cells, but no IFJV1 replication was detected in these cells (see Supplementary Figure S1). These results are in accordance with previously published results on jingmenviruses.

### 3.4. Virus Derived Small RNA Profiles

**Figure 3:**
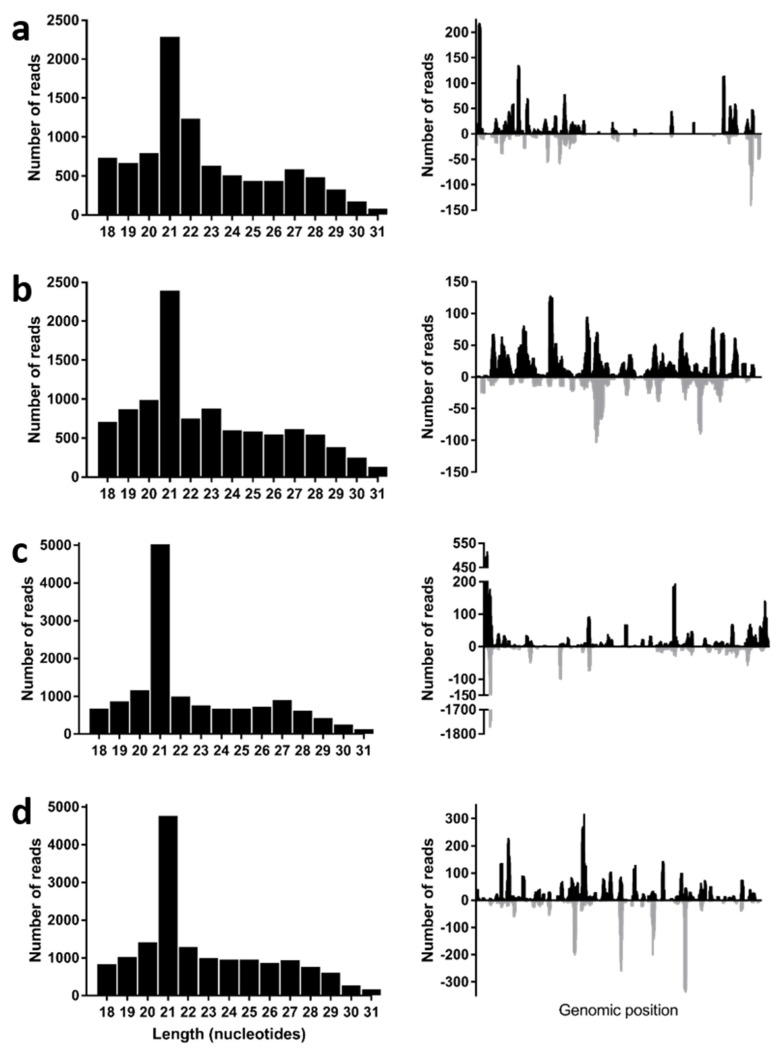
Length profile and distribution of IFJV1−derived sRNAs. The virus−derived sRNA length profiles are on the left: (**a**) segment 1; (**b**) segment 2; (**c**) segment 3; (**d**) segment 4, and the distribution of 21 nt-long IFJV1-derived sRNA mapped back to the virus in positive (black) and negative (grey) sense nucleotide sequences are on the right.

Despite its inability to replicate in classical in vitro laboratory models, we analyzed the sRNA in one of the positive pools of 20 SG from starved larvae to determine if the virus was replicating in its soldier fly hosts. During a virus infection in arthropods, virus-related double-stranded RNA (dsRNA) triggers the activity of the host RNAi’s responses and the host riboendonuclease III enzyme Dicer-2 cleaves this dsRNA into virus-derived, small interfering RNAs (vsiRNAs), which are 19–22 nt in length [20]. These vsiRNAs are then loaded into the RNA-induced silencing complex, where they target RNA molecules through complementarity, reduce the virus gene transcription and, ultimately, virus replication. For insect viruses, the vsiRNAs display a sharp peak in 21 nt, are symmetrically distributed throughout the viral genome, and map to both strands (positive and negative) [21,22,23,24]. Therefore, after selecting only the reads between 18 and 30 nt in length, we mapped the sRNA libraries to the three IFJV1 segment sequences and generated the size distribution graphs (Figure 3). We found that the virus-derived sRNAs displayed a peak in size at 21 nt in length and, when the 21 nt reads were mapped back to each segment, we observed that most of the four sequences were covered by the sRNA reads on both strands. This suggests that IFJV1 was replicating actively in its soldier fly host and activated its immune response.

## 4. Discussion

Here, we report the discovery and identification of a novel jingmenvirus in Australian sugarcane soldier flies. This is the first report of a jingmenvirus in Oceania, which extends the distribution of jingmenviruses to all continents except Antarctica. This ubiquitous distribution highlights that jingmenviruses should be studied and their emerging potential characterized. Moreover, the close association between *I. flavus* larvae (which is the stage in which the virus was found) and sugarcane leads us to question whether the virus replicates within the plants and/or interacts with it in another way. This consideration is all the more relevant, given that the Wuhan aphid virus 2 was detected both in an insect (*Hyalopterus pruni*) and in a plant (*Pisum sativum*), with the two strains displaying high levels of sequence similarity [25,26]. The effect of the virus in *I. flavus* and its potential as a biological control agent of the pest need to be investigated. Unfortunately, the absence of a laboratory replication model for IFJV1 at this stage prevents the in-depth characterization of its tissue tropism in *I. flavus*, its modes of transmission, its pathogenicity both in *I. flavus* and sugarcane, and its potential for interference with viruses co-circulating in soldier flies and sugarcane. 

Interestingly, the IFJV1 sequences clustered with the two mosquito-associated jingmenviruses: the Guaico Culex virus and the Mole Culex virus, in both phylogenies, based on NSP1 and NSP2 [17,19]. However, while mosquitoes and soldier flies both belong to the *Diptera* family, other jingmenviruses that were detected in dipteran insects, such as Drosophilae or Culicoides, do not cluster together. Furthermore, the jingmenviruses that have been detected in hemipteran hosts do not cluster together, nor with those found in the closely related families *Thysanoptera* and *Psocodea* [27]. The phylogenetic organization of insect-related jingmenviruses, therefore, does not follow the phylogenetic organization of their identified hosts [27]. This observation is corroborated by the presence of jingmenviruses within the phylogeny, detected in non-insect arthropods from two other classes: Louisiana crawfish (*Procambarus clarkia;* Malacostraca) and small wood scorpions (*Euscorpius sicanus;* Arachnida), as well as in organisms from the Fungal (*Erysiphe necator* and *Plasmopara viticola*) and Plant (*Pisum sativum*) kingdoms [26,28,29,30,31]. This lack of correlation between the virus’s and host’s phylogenies suggests that the viruses have not co-evolved with their hosts. Since most of the jingmenvirus sequences originate from metagenomics studies, this observed phenomenon could be due to an incorrect host assignment, since the method would not differentiate sequences from an insect, an insect’s previous meal, a contaminating parasite, or a fungus. Another reason for the observed discrepancy between the host’s and virus’s phylogenies could be the existence of a reservoir for these viruses outside of the *Insecta* class; for example, in plants or fungi, as suggested above. The viruses could be transmitted to the insect hosts from this reservoir, enabling independent evolution. In any case, jingmenviruses need to be studied further to elucidate their ecology and their modes of transmission. 

Unfortunately, we were not able to further investigate these considerations, since the virus could not be cultured stably in vitro in any cell line. Our results show that IFJV1 displayed limited replication at the first two passages in insect cells, but that this replication could not be sustained past that stage. In vertebrate cells, IFJV1 replication could not be detected even in the first passage, suggesting it could be even more restricted in vertebrates. Isolation was also attempted for the prototype species of jingmenviruses: JMTV, among others, but all were unsuccessful or replication was shown to be limited to the first couple of passages [8,12,13,14,15,16,17]. The mechanisms involved in this replication restriction in laboratory models are not yet clear [5,12,13]. Since we identified other viruses in the salivary gland sequencing data, it could be possible that the lack of IFJV1 replication observed was due to an interfering co-infecting virus. However, we had no evidence of an actively replicating virus in the inoculated cells (as no cytopathic effect was evident). Moreover, we attempted the isolation of another virus detected in the sequencing data (these data were not shown) and could not detect the viral RNA past the initial inoculation, suggesting that the detection of IFJV1 after only two passages is specific to this virus. In addition, we showed that IFJV1 can elicit an immune response from its host via the RNAi pathways by analyzing the sRNA in *Inopus flavus* homogenates. However, the C6/36 cell line has a dysfunctional RNAi response, so this mechanism is unlikely to prevent IFJV1 replication in vitro in these cells [32]. The presence of an IFJV1-specific sRNA response is, however, proof that the virus is replication-competent in its host, since vsiRNAs are produced in the presence of abundant dsRNA, which only occurs in the form of replicative intermediates for actively replicating +ssRNA viruses [33]. These data, therefore, show that the lack of replication observed in vitro is due to an inappropriate model rather than a replication-incompetent virus. The sRNA data also demonstrate that IFJV1 is indeed an *I. flavus* virus and that this host has been assigned correctly.

Overall, our study increases the knowledge on jingmenviruses by adding a new member from a new continent as well as a new host to this sub-genus. This report also demonstrates the importance of developing a stable laboratory model for the replication of jingmenviruses, to ensure their thorough characterization and the evaluation of their potential to emerge as insect, plant, or vertebrate pathogens.

## Figures and Tables

**Table 1:** Primers designed to detect IFJV1.

Segment	ORF	Direction	Name	Sequence	Product Size
1	NSP1	Forward	IFJV1 seg1 1F	CCTGGAAGATGTATGGTGTGATTGG	514 bp
		Reverse	IFJV1 seg1 1R	GCTCCTTTCCCTGTTCTATCTTGG	
3	NSP2	Forward	IFJV1 seg3 1F	GGAAGACTCAAACAGAATCTCATGC	542 bp
		Reverse	IFJV1 seg3 1R	GGTACTTCGCATGTCACATGC	
4	VP2	Forward	IFJV1 seg4 1F	GGTAGCAAGTTACAAGATGG	523 bp
		Reverse	IFJV1 seg4 1R	CATACACAACATCTCCATATGTGTGG	

## Data Availability

Deep sequencing raw data have been deposited in the National Centre for Biotechnology Information’s (NCBI’s) Gene Expression Omnibus (GEO) and are accessible through GEO series accession number: GSE127658. The IFJV1 genomic sequences have been deposited on Genbank and have been assigned the following accession numbers: OM869459-OM869462.

## Supplementary Materials

The following supporting information can be downloaded at: https://www.mdpi.com/article/10.3390/v14061140/s1, Figure S1: Replication of IFJV1 in insect and vertebrate cell lines.
