# Supplementary material for: Discovery of a Novel Jingmenvirus in Australian Sugarcane Soldier Fly (Inopus flavus) Larvae
Source: Viruses. 2022 May 25;14(6):1140. doi: 10.3390/v14061140 (PMC9229714; doi:10.3390/v14061140)
Supplement: Supplementary file 1 [file viruses-14-01140-s001.zip › viruses-1658217-supplementary.pdf]

Supplementary Materials

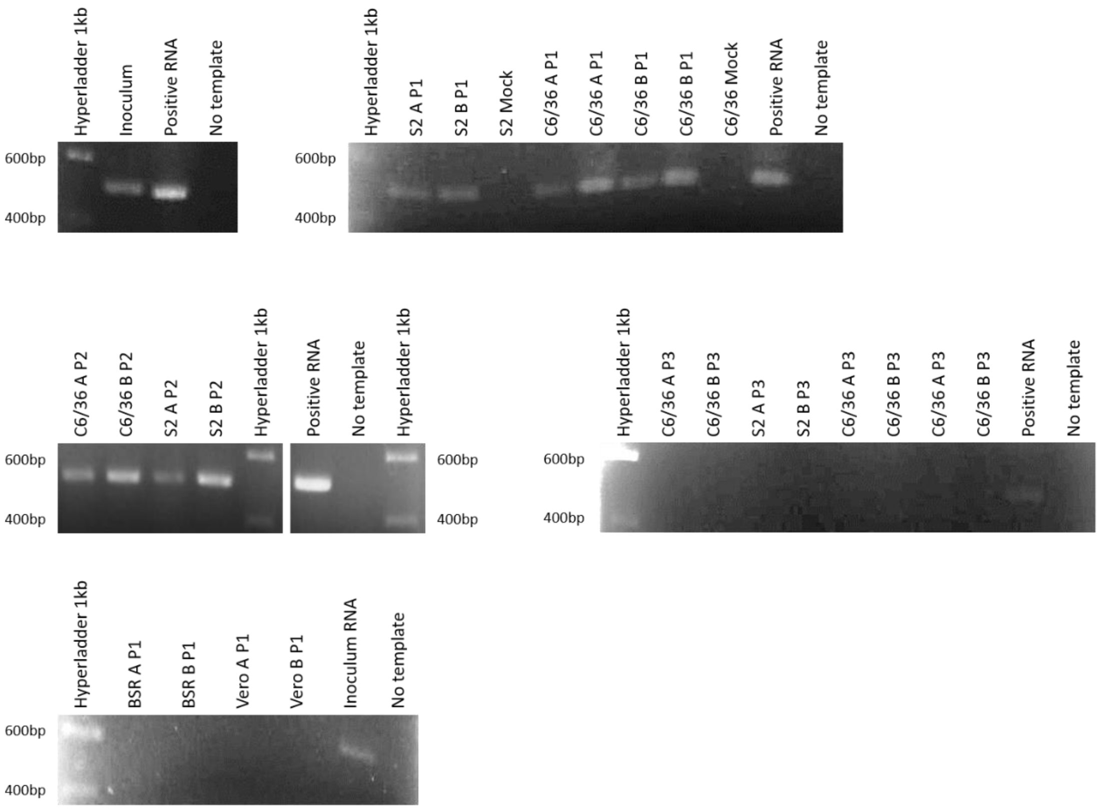

Figure S1. Replication of IFJV1 in insect and vertebrate cell lines. IFJV1 displayed limited replication at the first two passages in insect cells but this replication could not be sustained past that stage. In vertebrate cells, IFJV1 replication could not be detected even in the first passage, suggesting it could be even more restricted in vertebrates. P1-3: Passage 1-3. C6/36: *Aedes albopictus* cell line; S2: *Drosophila melanogaster* cell line; BSR: a cell line from Golden hamster *Mesocricetus auratus*; Vero: a cell line derived from African green monkey (*Chlorocebus* sp.).
